# Amplifying the electrochemical footprint of <1000 molecules in a dissolving microdroplet†

**DOI:** 10.1039/d4an00504j

**Published:** 2024-05-17

**Authors:** James H. Nguyen, Ashutosh Rana, Jeffrey E. Dick

**Affiliations:** a Department of Chemistry, Purdue University West Lafayette IN 47907 USA jdick@purdue.edu; b Elmore Family School of Electrical and Computer Engineering, Purdue University West Lafayette IN 47907 USA

## Abstract

The ability of analytical strategies to detect and positively identify molecules under extremely dilute conditions is important for the growth and expansion of analytical techniques and instrumentation. At present, few measurement science techniques can robustly approach the measurement of just a few thousand molecules. Here, we present an electrochemical platform for the detection and positive identification of fewer than 1000 molecules of decamethylferrocene ((Cp*)_2_Fe^II^). We achieve this remarkable detection threshold by trapping (Cp*)_2_Fe^II^ in a 1,2-dichloroethane microdroplet, which is allowed to dissolve into an aqueous continuous phase while on a gold microelectrode (radius ∼6.25 μm). Because electrochemistry is not sensitive enough to observe the charge of less than 1000 molecules, we dissolved μM amounts hexacyanoferrate(iii) in the aqueous continuous phase. The biphasic reaction between hexacyanoferrate(iii) and Cp_2_*(Fe)^II^ allows for a feedback loop when the microelectrode is biased sufficiently negative to reduce Cp_2_*(Fe)^III^. This feedback loop, a typical EC′ catalytic mechanism, amplifies the electrochemical signal of Cp_2_*(Fe)^II^ when the droplet is of small enough dimensions for feedback to occur. Our results demonstrate that clever biphasic reactions can be coupled with dissolving microdroplets to access extremely low limits of quantitation in electroanalysis.

## Abstract 2

We present an electrochemical platform for the detection and identification of <1000 molecules through an EC' mechanism. The EC' catalytic mechanism allows for a feedback loop mechanism which amplifies the electrochemical signal of Cp_2_*(Fe)^II^.

## Graphical abstract

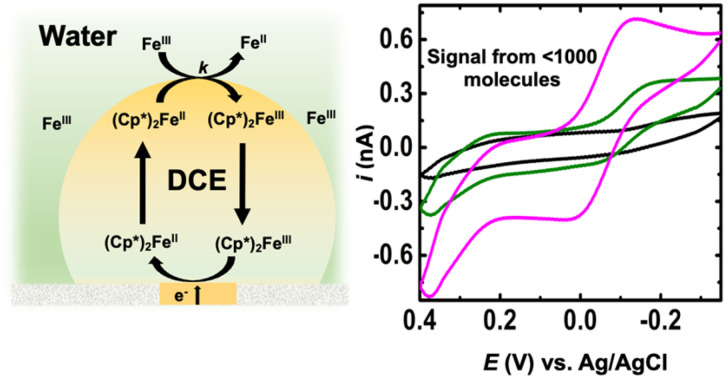

## Introduction

Analytical strategies for trace detection have been a crucial area of research, with advancements in instrumentation enabling detection at extremely low concentrations. In addition to sophisticated instruments, various techniques have emerged to aid in the detection of low concentrations. One notable approach involves leveraging electrochemical analysis coupled with amplification methods, offering a novel strategy for trace detection within the framework of a well-established method. Gas chromatography-mass spectrometry (GC-MS) is one such technique that can detect trace amounts of redox analyte by analyzing volatile compounds (1.80 μg L^−1^).^1^ Liquid chromatography-mass spectrometry (LC-MS) is another chromatography technique capable of detecting low concentrations through liquid separation (0.741 ng mL^−1^).^2,3^ Spectroscopy methods can also be employed for trace detection. Atomic absorption spectroscopy (AAS), for instance, detects trace heavy metals by measuring the absorption of light by atoms in a sample (0.05 μg L^−1^).^4,5^ Fluorescence spectroscopy, on the other hand, measures the fluorescence emitted by a sample when exposed to ultraviolet light (0.48 pM).^6^ Inductively coupled plasma-mass spectrometry (ICP-MS) is another powerful analytical technique used for ultra-sensitive detection of trace elements by exciting a sample with plasma (1 nmol L^−1^).^7,8^ Overall, sensitive detection of low concentrations of redox analytes is crucial in analytical chemistry for the development of novel analytical strategies. As trace detection becomes more stringent and the identification of target analytes becomes more challenging, enhancing sensor sensitivity becomes increasingly vital. Signal amplification strategies are crucial for enhancing the sensitivity of sensors, particularly in the detection of trace contaminants.^9^ One widely used approach is enzyme-catalyzed signal amplification, where enzyme-catalyzed reactions induce electrochemical responses at the electrode surface.^10,11^ Nanomaterial-based signal amplification is another effective strategy, harnessing nanomaterials with superior electrochemical properties such as conductivity and catalytic activity.^12,13^ Additionally, nucleic acid-based signal amplification, targeting DNA to generate electrical signals, is commonly utilized in biosensor construction for its high sensitivity.^14–16^ Redox cycling, another signal amplification technique, involves the repeated oxidation and reduction of a redox species between two parallel electrodes. This continuous alternation between reduction and oxidation generates a current of sufficient magnitude to be detected, resulting in charge amplification. After Bard and co-workers initial report, Lemay's group strengthened the strategy of redox cycling by lithographically fabricating nanogaps.^17–19^ Moreover, scanning electrochemical microscopy (SECM) has been employed alongside redox cycling to amplify electrochemical signals. When the SECM probe approaches the redox mediator, it initiates a positive feedback loop or cyclic regeneration of the mediator at the interface, thereby amplifying the current.^20^ These strategies collectively contribute to significant improvements in sensor sensitivity, enabling the detection of trace compounds with greater precision and accuracy.

Droplet-based sensors utilize small liquid droplets to detect low concentrations of specific analytes.^21^ This technology, facilitated by droplet-based microfluidics, enables manipulation of miniscule volumes (fL-aL) within these droplets.^22^ Acting as microreactors, the droplets are encapsulated by an immiscible phase, providing protection and facilitating manipulation. Specialized variants, such as evaporating or dissolving droplets, concentrate the analyte for detection using electrochemical techniques.^23^

Droplet-based sensing using evaporation, particularly when combined with surface-enhanced Raman spectroscopy, has demonstrated significant promise.^24,25^ Our group recently demonstrated an electrochemical methodology for detecting trace analytes using droplet-based sensing involving droplet dissolution.^26–30^

It was demonstrated that the dissolution of microdroplets effectively concentrates redox-active analytes in a confined volume, enabling their detection through methods like cyclic voltammetry, even at ultra-low concentrations (sub-nM, roughly 10^6^ molecules) of redox-active analytes. In this work, we demonstrate how the previously reported methodology can be coupled with a signal amplification strategy to further reduce the limit of detection or enhance the ability to detect an electrochemical response from thousands of molecules. We report on the utilization of dissolving droplet electroanalysis in conjunction with EC′ reaction (which denotes an electron-transfer reaction (E) coupled with a chemical reaction (C) where the species involved in the electron transfer reaction is regenerated) at the liquid–liquid interface of an oil droplet submerged in a bulk aqueous phase. A typical reaction pathway for EC′ is shown in eqn (1) and (2) below:

1 A + e^−^ → B

2 B + X → A + Y

where E represents the electron-transfer reaction at the electrode surface, involving the reduction of A to B. Subsequently, B is involved in a chemical reaction (C) with some species X present in the solution to generate species A and Y.^31,32^ Dichloroethane (DCE) droplets spiked with varying amounts of decamethyl ferrocene ((Cp*)_2_Fe^II^) were carefully positioned atop a 6.25 μm radius gold ultra-microelectrode and allowed to dissolve in a bulk aqueous phase comprising 10 mM NaClO_4_ as a phase-transfer agent and varying concentrations of potassium ferricyanide (K_3_[Fe(CN)_6_]) to trigger the EC′ mechanism (*vide infra*). Using this platform, we demonstrate the detection of (Cp*)_2_Fe^II^ at sub-picomolar (sub-pM) concentrations in the DCE phase, outlining an electrochemical footprint for detecting less than 1000 molecules. Detection of (Cp*)_2_FeII without the addition of the EC′ reaction showed a limit of detection (LOD) at the sub-nanomolar (sub-nM) level. Overall, this work unveils an electrochemical technique for detecting fewer than 1000 molecules, representing a significant advancement in electrochemical analysis. The outcomes of this study hold significant promise for advancing analytical chemistry and sensor development, offering valuable insights that could fuel future progress in these areas.

## Materials and methods

All aqueous solutions were prepared using ultra-pure deionized water with a resistivity of 18.2 MΩ cm, sourced from a GenPure water purification system manufactured by Millipore. The organic solvent 1,2-dichloroethane, 99.8% purity (DCE) was acquired from Sigma Aldrich. The salts for all the experiments; decamethyl ferrocene ((Cp*)_2_Fe^(II)^), was obtained from Sigma Aldrich. Potassium Ferricyanide (K_3_[Fe(CN)_6_]) was obtained from Thermo Scientific Chemicals. Sodium Perchlorate (NaClO_4_) was obtained from Sigma Aldrich. All reagents were of analytical grade and were used without any additional purification. Prior to experimentation, the glassware underwent meticulous cleaning using mQ water, followed by acetone (99.9%, Sigma-Aldrich), and finally with the relevant solvent for each solution. Gold working electrodes with a diameter of 12.5 μm were obtained from CH Instruments, while the Ag/AgCl reference electrode in 1 M KCl, was purchased from the same supplier and was employed as the counter/reference electrode. Before usage, the working electrodes were polished with a 0.05 μm alumina powder suspension (Electron Microscopy Sciences) on micro-cloth polishing pad (Buehler) using water. Subsequently, they underwent a cleaning process with piranha solution, which was a mixture of concentrated sulfuric acid with 30% hydrogen peroxide in a 3 : 1 ratio, to ensure thorough purification. The lab-made electrochemical cell, constructed out of Teflon, was also carefully cleaned using Piranha solution to eliminate any potential impurities. Microinjection experiments were performed using a micro-injector (FemtoJet 4i Eppendorf) and microinjection capillary tips with an orifice diameter of 10 μm (Eppendorf Femtotips). The position of the microinjector was controlled using an XYZ micro-positioning system (InjectMan 4) and monitored with an optical microscope equipped with a high-resolution sCMOS camera (C15440 Orca Fusion BT). All electrochemical experiments were conducted using a CHI 6284E potentiostat (CH Instruments). The reference electrode was placed in a separate compartment containing 1 M KCl and was connected to the cell through a salt bridge. The salt bridge was created by filling a glass tube with 3% agarose (99.9%, Sigma-Aldrich) containing 1 M potassium chloride.

## Results and discussion

**Fig. 1:**
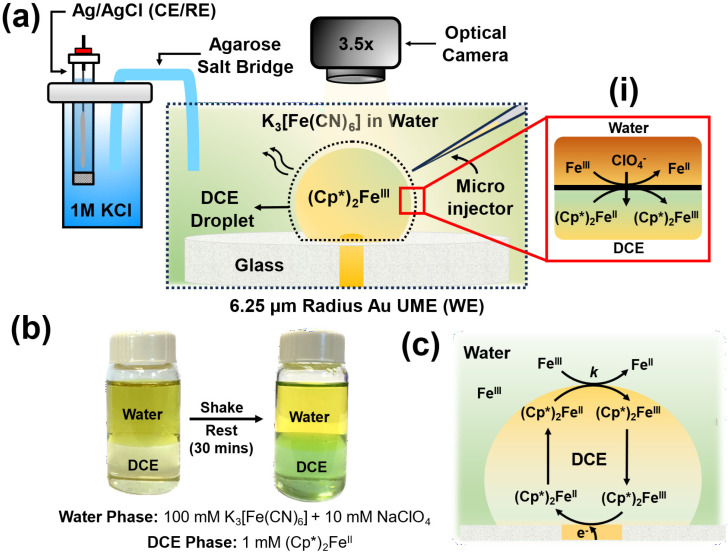
(a) Experimental setup illustrating the components: (Cp*)_2_Fe^II^ in a DCE droplet, K_3_[Fe(CN)_6_] in the aqueous bulk phase. The cell comprises an Au UME, Microinjector, and an agarose salt bridge connecting to a 1 M KCl reservoir with the CE/RE Ag/AgCl. Experiment monitoring is facilitated by an optical camera. (i) Chemical reaction at the oil|water interface, where Fe(CN)_6_^3−^ denoted as Fe^III^ is reduced to Fe(CN)_6_^3−^ denoted as Fe^2+^, and (Cp*)_2_Fe^II^ oxidizes to (Cp*)_2_Fe^III^, with ClO_4_^−^ transitioning between the phases to maintain electroneutrality. (b) Shake-flask experiment where the water phase contains 100 mM K_3_[Fe(CN)_6_] and 10 mM NaClO_4_, while the DCE phase contains 1 mM (Cp*)_2_Fe^II^. After shaking and resting for 30 minutes, the DCE phase changes to a green color, indicating oxidation of (Cp*)_2_Fe^II^. (c) Mechanism of the EC′ reaction in a droplet.

The experimental setup is illustrated in Fig. 1(a). In a typical experiment, a DCE droplet spiked with (Cp*)_2_Fe^(II)^ is injected and positioned onto a 6.25 μm radius Au UME using a microinjector. The DCE droplet spontaneously dissolves into the bulk aqueous phase when the concentration of DCE in the bulk aqueous phase is lower than the solubility limit of DCE in water (0.869 g per 100 mL at 20 °C). The aqueous phase comprised varying concentrations of K_3_[Fe(CN)_6_] across experiments, along with 10 mM NaClO_4_. As the droplet dissolves, electrochemical measurements are conducted concurrently with real-time monitoring of the droplet's geometry using a high-resolution camera. Micrographs are acquired in bright field mode using diffused white light illumination. All electrochemical measurements were conducted using a two-electrode setup, with the Au UME serving as the working electrode and Ag/AgCl in 1 M KCl, connected by an agarose salt bridge, serving as the reference/counter electrode. Top of form: The droplet initially contains only (Cp*)_2_Fe^II^ molecules, which can spontaneously react with Fe(CN)_6_^3−^ (shown as Fe^3+^) to get oxidised to (Cp*)_2_Fe^III^ at the oil–water interface, as shown in inset (i) in Fig. 1(a). It's important to note that this reaction occurs spontaneously and doesn't require the application of any form of bias (potential/current) to the electrode. Such a biphasic reaction requires the partitioning of ClO_4_^−^ ions from the aqueous phase to the oil phase to maintain electroneutrality. Top of form: To validate the occurrence of the biphasic reaction depicted in inset (i) of Fig. 1(a), shake-flask experiments were conducted using equal volumes of the two phases, as illustrated in Fig. 1(b).^33,34^ Bottom of form: The water phase initially contained 100 mM K_3_[Fe(CN)_6_] and 10 mM NaClO_4_, resulting in a dark yellow coloration, while the oil phase contained 1 mM (Cp*)_2_Fe^II^, appearing pale yellow in color. The vial was shaken until an emulsion was visually observed and then left to rest for 30 minutes. After this period, the oil phase turned greenish, indicating the conversion of (Cp*)_2_Fe^II^ to (Cp*)_2_Fe^III^, thus demonstrating that the DCE phase is primarily comprised of (Cp*)_2_Fe^III^ molecules. Concurrently, there was a change in the coloration of the water phase due to the reduction of Fe(CN)_6_^3−^ to Fe(CN)_6_^2−^. These reactions simply follow from the reaction mechanism depicted in inset (i) of Fig. 1(a).

The concept of using this strategy at the oil–water interface to amplify the voltametric response under a dissolving oil droplet configuration is depicted in Fig. 1(c). The nature of the reaction occurring within the system is similar to the EC′ reaction discussed earlier in Fig. 1, where A represents (Cp*)_2_Fe^III^, which reduces to (Cp*)_2_Fe^II^ (B) at the electrode surface. Subsequently, (Cp*)_2_Fe^II^ chemically reacts with Fe(CN)_6_^3−^ (X) at the oil–water interface to produce (Cp*)_2_Fe^III^ (A) and Fe(CN)_6_^2−^ (Y). For instance, during voltametric sweep, where (Cp*)_2_Fe^III^ is converted to (Cp*)_2_Fe^II^, the generated (Cp*)_2_Fe^II^ molecules can be converted back to (Cp*)_2_Fe^III^ at the oil–water interface and be incident on the electrode again. This sets up a feedback loop which can enhance the observed signal. The following discussion has been segmented into three sections focusing primarily on control experiments in the absence of any amplification strategy, experimental considerations associated with observing EC′ voltametric response in the presence of the EC′ and amplifying the electrochemical footprint for ultra-low concentrations of (Cp*)_2_Fe^II^ (sub-pM). These types of experiments are reminiscent of nanogap and thin-layer experiments.^35–38^ However, instead of using another electrode to perform the reverse redox reaction, we rely on the biphasic reaction at the liquid|liquid interface.

• Control experiments in the absence of amplification strategy.

**Fig. 2:**
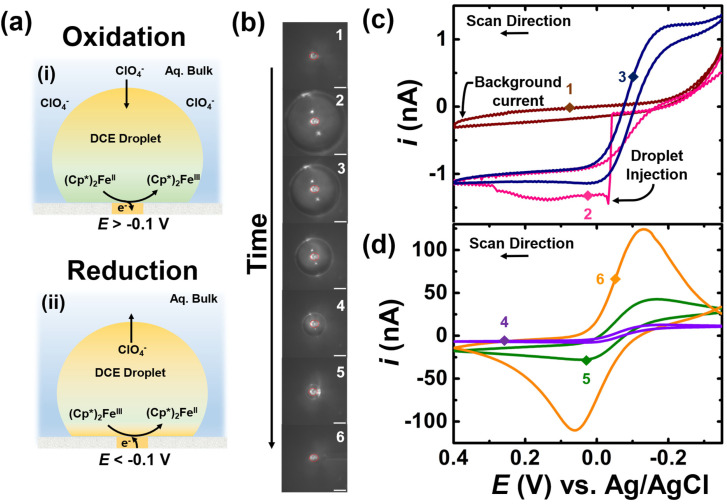
(a) Oxidation (i) and reduction (ii) schematic illustrating the partitioning of ClO_4_^−^ ions across the oil|water interface. (b) Optical micrographs depict the decreasing droplet size over time, with a scale bar of 25 μm. (c) and (d) Cyclic voltammograms recorded from before droplet injection to complete dissolution. The aqueous bulk phase contains 10 mM NaClO_4_ in water and DCE droplet is spiked with of 0.5 mM (Cp*)_2_Fe^II^. Six CVs were taken at different time points to demonstrate the electrochemical response at specific droplet sizes. The marked points on the cyclic voltammogram indicate the exact time when the frames in (b) were recorded.

The experimental result for the control experiment in the absence of K_3_[Fe(CN)_6_] species in the bulk is shown in Fig. 2. The aqueous bulk phase contains 10 mM NaClO_4_ in water and DCE droplet is spiked with of 0.5 mM (Cp*)_2_Fe^II^. The apparent standard potential for the redox couple (Cp*)_2_Fe^III^/(Cp*)_2_Fe^II^ is observed at −0.1 V *vs.* Ag/AgCl.^39^ The ClO_4_^−^ species in the aqueous phase allow for maintaining electroneutrality inside the droplet. For instance, applying a potential more positive than −0.1 V allows for the oxidation of (Cp*)_2_Fe^II^ to couple (Cp*)_2_Fe^III^, which involves partitioning of the ClO_4_^−^ ion into the DCE phase. On the other hand, applying any potential more negative than −0.1 V results in the reduction of (Cp*)_2_Fe^III^ to (Cp*)_2_Fe^II^, which involves partitioning of the ClO_4_^−^ anion from the DCE phase into the water phase. The overall charge-balance mechanism is shown in inset (i) and (ii) of Fig. 2(a). The DCE droplet containing (Cp*)_2_Fe^II^ is injected onto the electrode surface and spontaneously dissolves over time. The optical micrographs acquired during the dissolution of the droplet are shown in Fig. 2(b). The position of the electrode is marked with a solid red circle at the center of each micrograph. The initial droplet size was measured to be 120 μm based on micrograph 2 shown in panel (b) of Fig. 2. At this time, we cannot control the dissolution dynamics on a droplet-by-droplet basis. We have previously studied the forces that are at play that govern the droplet dissolution dynamics.^40^ Given the differential equations that dictate droplet dissolution, we can estimate a droplet size as a function of time; however, we lack the sensitivity to electrochemically confirm this. The potential of the UME was continuously scanned between an initial potential of −0.35 V to 0.4 V *vs.* Ag/AgCl at a scan rate of 0.2 V s^−1^ to observe the effect of droplet dissolution on the electrochemical response for the redox couple confined in the droplet. A total of 81 voltammograms were recorded between micrograph 1 and 6 in panel (b) of Fig. 2. Out of the total voltammograms recorded, six are presented in Fig. 2(c) and (d). The brown, pink, and navy curves represent CV1, CV2, and CV25, respectively, while the purple, green, and orange curves represent CV49, CV77, and CV81. The numbered points on the voltammograms correspond to the numbering on the micrographs in panel (b), indicating the size of the droplet at that point. Prior to the injection of the droplet, a background current was recorded (optical image 1, brown voltammogram in panel (c) of Fig. 2), indicating the absence of any redox activity. The signal arises only from the charge and discharge of the electrochemical double layer.

The injection of the droplet appears as a sharp increase in current from the background current (optical image 2, pink voltammogram) indicating electrochemical response or oxidation of (Cp*)_2_Fe^II^ to (Cp*)_2_Fe^III^ confined in the droplet. When the droplet size is large compared to the electrode, a sigmoid-shaped voltammogram is initially observed (optical image 3, blue voltammogram), representative of a bulk-like condition. As the droplet shrinks over time, the magnitude of observed current in the voltammograms increases due to the spontaneous enrichment in the concentration of the redox analytes confined in the droplet.^39^ The voltammograms transition from an initial sigmoid (navy curve) to a duck-shape (green curve) and finally transition into a Gaussian pair of peaks indicating thin-layer conditions (orange curve). Note that the voltammogram recorded subsequent to the orange curve (CV 81 and optical micrograph 6 in panel (b) of Fig. 2) shows no redox activity due to the complete dissolution of the droplet. Overall, these results highlight two main findings: firstly, there is an enrichment in the concentration of redox analytes confined in the droplet, and secondly, the shape of the voltammograms changes distinctly as the droplet shrinks.

• Experimental consideration for witnessing EC′ on the voltammetric response.

**Fig. 3:**
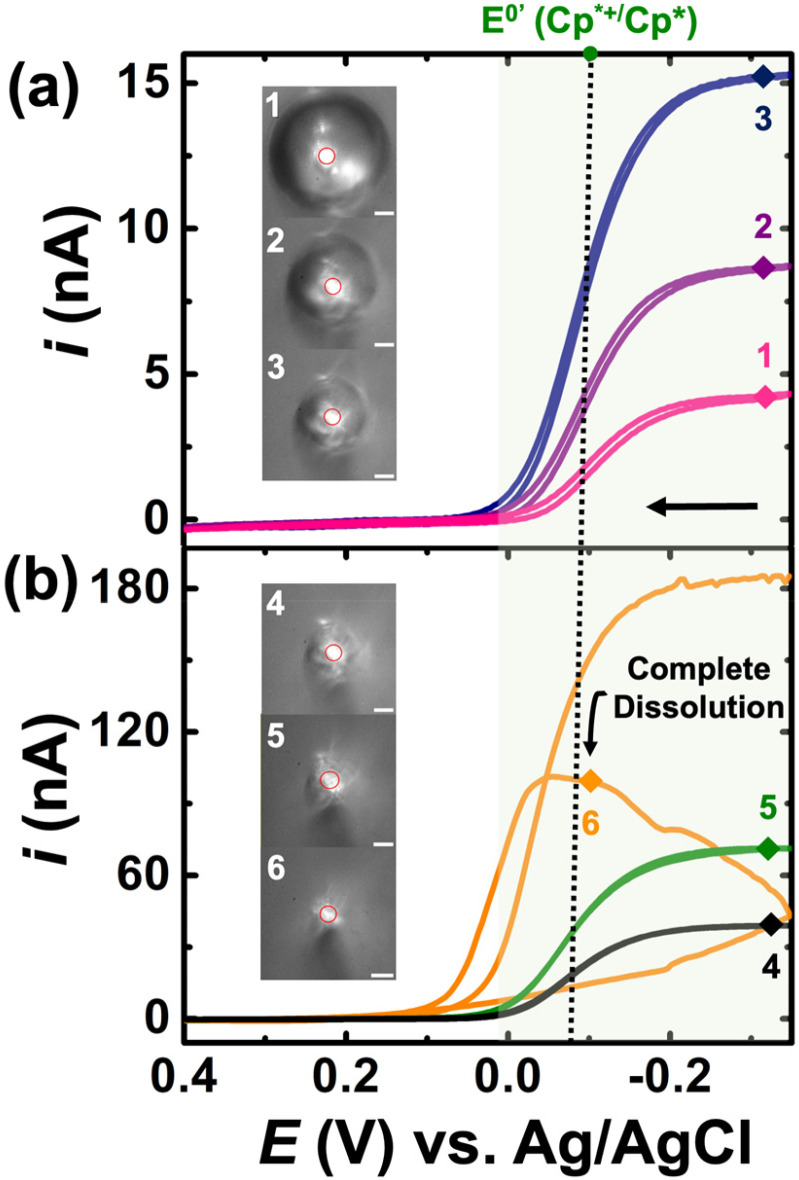
Cyclic voltammograms ((a) and (b)) recorded during this dissolution of DCE droplet containing 0.5 mM (Cp*)_2_Fe^II^. The aqueous bulk phase contains 100 mM K_3_[Fe(CN)_6_] and 10 mM NaClO_4_ in water. The dotted line marks the apparent standard potential for the redox couple (Cp*)_2_Fe^III^/(Cp*)_2_Fe^II^. The numbered points on the cyclic voltammogram indicate the exact time at which the numbered micrographs were recoded. The scale bar for the micrographs is 20 μm.

For the experiments detailed in this section, a similar experimental setup was used as detailed previously, but K_3_[Fe(CN)_6_] was introduced into the bulk aqueous phase. The DCE droplet was spiked with 0.5 mM Cp_2_*(Fe)^II^ and the aqueous phase comprised of 100 mM K_3_[Fe(CN)_6_] + 10 mM NaClO_4_. The droplet dissolution experiment started with adding 1.4 mL of 10 mM NaClO_4_ into the electrochemical cell. A DCE droplet with 0.5 mM Cp_2_*(Fe)^II^ was then injected and positioned onto the Au disk. After injection, 1.4 mL of a solution of 200 mM of K_3_[Fe(CN)_6_] + 10 mM of NaClO_4_ was added into the same cell equaling the desired concentration K_3_[Fe(CN)_6_] in the bulk phase. It is essential to use such a strategy to avoid interference in the redox activity of Cp* from of K_3_[Fe(CN)_6_] (see Fig. S1†). This is discussed in detail in the latter half of the section. The droplet dissolution experiment began by adding 1.4 mL of 10 mM NaClO4 into the electrochemical cell. Subsequently, a DCE droplet containing 0.5 mM Cp_2_*(Fe)^II^ was injected and positioned onto the Au disk electrode. After injection, 1.4 mL of a solution containing 200 mM of K_3_[Fe(CN)_6_] + 10 mM of NaClO_4_ was added into the same cell to achieve the desired concentration of K_3_[Fe(CN)_6_]in the bulk phase. This strategy was employed to prevent interference in the redox activity of (Cp*)_2_Fe^II^ from K_3_[Fe(CN)_6_], as discussed in detail later in the section. The experimental results are shown in Fig. 3(a) and (b). Note that the micrographs and voltammograms presented are only after spiking the solution with K_3_[Fe(CN)_6_] solution. Cyclic voltammograms were recorded in a potential window of −0.35 V to 0.4 V at a scan rate of 0.2 V s^−1^. Similar to the results presented previously, the redox activity is observed at the apparent standard potential for the redox couple (Cp*)_2_Fe^III^/(Cp*)_2_Fe^II^ (−0.1 V *vs.* Ag/AgCl). This potential is represented by a dotted line and a green shade showing (Cp*)_2_Fe^III^/(Cp*)_2_Fe^II^ redox activity.

A total of 41 voltammograms were acquired between micrographs 1 and 6 in Fig. 3(a) and (b). In this series, pink, purple, and navy represent CV1, CV14, and CV24, while black, green, and yellow represent CV34, CV39, and CV40. The voltammograms are labeled 1–6 to match the micrograph numbering, reflecting the droplet size at each stage. The initial droplet size in micrograph 1 has a radius of 60 μm. A noticeable alteration in the electrochemical response occurs with the introduction of K_3_[Fe(CN)_6_] in the aqueous phase. In its absence, the voltammograms evolve from an initial sigmoid shape to a duck-shaped curve and ultimately to a Gaussian pair of peaks. Conversely, in the presence of K_3_[Fe(CN)_6_], only sigmoid-shaped voltammograms are evident in Fig. 3, with increasing steady state currents observed throughout the dissolution of the DCE droplet. These findings can be readily interpreted in light of the EC′ mechanism shown in Fig. 1(c). At any given moment within the droplet, the ferrocenyl redox molecules exist in their oxidized form, (Cp*)_2_Fe^III^, due to the reaction occurring at the oil|water interface. Consequently, the voltammograms depicted in Fig. 3 demonstrate the reduction of (Cp*)_2_Fe^III^ to (Cp*)_2_Fe^II^, with any generated (Cp*)_2_Fe^II^ subsequently converted back to (Cp*)_2_Fe^III^ at the oil–water interface. At this point, it's crucial to acknowledge that this strategy can significantly amplify the voltammetric signal for the confined redox molecules within the droplet, especially as the droplet accesses minute volumes. This amplification arises because when the droplet size is comparable to that of the electrode, all confined redox molecules can respond to the applied bias at the electrode surface during a voltammetric sweep. Under these conditions, any Cp_2_*(Fe)^II^ generated at the electrode surface can be converted back to Cp_2_*(Fe)^III^ at the oil|water interface and incident at the electrode surface during the same voltammetric sweep, thereby amplifying the signal. Note that the time the droplet accesses tiny volumes is extremely short-lived due to the increasing dissolution rate at smaller volumes. Therefore, our ability to detect the amplified signal also competes against the time-scale of the experiments, *i.e.*, the scan rate of the voltammetry experiments. Increasing the scan rate of the experiments can provide better time resolution, but it comes at the cost of increased capacitive current. One must be cognizant of the fact that droplets of the continuous phase (see Fig. S1†) can remain on the electrode when the microdroplet of DCE is pipetted on, as demonstrated in our previous work.^41^

• Detection of sub-pM levels of (Cp*)_2_Fe^II^.

This section demonstrates how the EC′ amplification strategy enables the observation of the electrochemical signature of fewer than 1000 molecules. It is crucial to note that electrochemistry alone lacks the sensitivity to detect the charge of less than 1000 molecules in any bulk measurement unless tailored with clever amplification strategies. At present, we are not able to quantify the amplification ratio for the EC′ reaction because this is highly dependent on the droplet's geometry on the microelectrode. Knowledge of the geometry from either optics or finite element modeling will help elucidate mechanistic aspects; however, in this paper, our main claim is the ability to amplify the footprint of just a few molecules. The preceding section demonstrated that the presence of K_3_[Fe(CN)_6_] in the bulk solution can obscure and interfere with the electrochemical response of (Cp*)_2_Fe^II/III^. Consequently, the concentration of K_3_[Fe(CN)_6_] was reduced from the 100 mM to 50 μM, and a spiking methodology was employed. However, detecting a concentration of 50 μM redox analyte is not feasible in bulk measurements, ensuring in the absence of a droplet we do not observe the redox activity of K_3_[Fe(CN)_6_]. In our studies, we used 50 μM of K_3_[Fe(CN)_6_]. We chose this concentration because voltammetry in the bulk could not be observed on a microelectrode at our relatively fast scan rates (∼1 V s^−1^). This scan rate was chosen because the amplification will be greatest when the droplet is smallest, and smaller droplets dissolve more rapidly. In fact, we were not able to see amplification at slow scan rates (∼0.01 V s^−1^). There are a few limiting factors with regard to our ability to electrochemically ‘visualize’ 1000 molecules: interaction of the analyte of interest with oxygen, analyte partitioning from the droplet to the continuous phase, and the droplet geometry (geometries that promote wetting and, thus, more of a nanogap will yield higher currents). At present, we do not purposefully control for these limitations, which are important for single molecule detection.

**Fig. 4:**
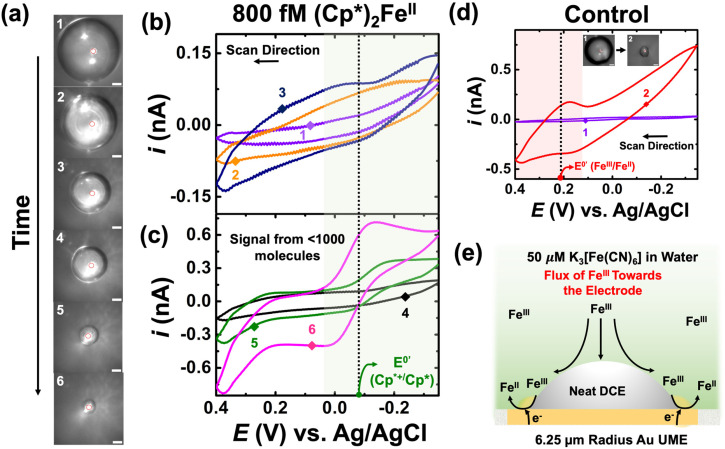
(a) Optical micrographs recorded during the dissolution of DCE droplet containing 800 fM of (Cp*)_2_Fe^II^ in an aqueous bulk phase containing 50 μM K_3_[Fe(CN)_6_] and 10 mM NaClO_4_. Cyclic voltammograms recorded during the dissolution of the DCE droplet shown in (a) are shown in (b) and (c). The voltammetric curves are numbered to correspond with the micrographs in (a), indicating the size of the droplet during the voltammetric sweep. (d) Optical micrographs and voltammogram for the control experiment in the absence of (Cp*)_2_Fe^II^ in the DCE droplet. (e) Schematic illustration for enhanced mass-transfer of K_3_[Fe(CN)_6_] to the electrode surface. Fe(CN)_6_^3−^ is represented as Fe^3+^. The scale bar for the micrographs is 20 μm.

Panel (a) of Fig. 4 depicts optical micrographs captured during the dissolution of the DCE droplet. The initial droplet size was measured to be 104 μm in radius, and it initially contained 800 fM of (Cp*)_2_Fe^II^. Using this information, one can directly calculate the amount of charge or molecules confined in the droplet. The initial droplet radius was measured to be 104 μm, and volume of the droplet was calculated to be 3 nL. When multiplied by the concentration (800 fM) and Avogadro's number (6.03 × 10^23^), it gives the number of molecules confined in the droplet. The value is found to be approximately 1000 molecules. Cyclic voltammograms were recorded between −0.35 V to 0.4 V at a scan rate of 1 V s^−1^. The standard apparent potential for the redox couple (Cp*)_2_Fe^III^/(Cp*)_2_Fe^II^ is represented by a dotted line and a green shade showing the redox activity in Fig. 4(b) and (c). A higher scan rate was chosen to increase temporal resolution, thereby enhancing the likelihood of observing the amplified voltammetric signal arising from the molecules confined in the droplet closer to its complete dissolution. Similar to the previous cases, the numbering on the micrographs is related to the numbering on the voltammograms shown in Fig. 4(b) and (c). Clearly, no redox activity is observed when the droplet size is large (micrographs 1, 2, 3 in Fig. 4(a)), as indicated by the voltammograms shown in Fig. 4(b). This is due to the very small signal-to-noise ratio, even with amplification. However, as the droplet accesses minuscule (sub-nL) volumes, there is a substantial amplification of the redox signal, allowing clear signals to arise from the molecules confined in the droplet (see green and purple voltammogram in Fig. 4(c)). It is essential to note the characteristics of the voltammogram, *i.e.*, the curve exhibits sigmoid characteristics, as expected based on the results discussed earlier in Fig. 3. We attribute the signal to <1000 molecules as there is some partitioning of the confined redox molecules from the DCE phase into the bulk aqueous phase, a phenomenon extensively discussed in our previously reported work. We gain more confidence that we are measuring molecules of (Cp*)_2_Fe^III^/(Cp*)_2_Fe^II^ because of the formal potential.

Control experiments were performed by injecting neat DCE droplets onto the electrode in a bulk aqueous phase containing 50 μM of K_3_[Fe(CN)_6_] and 10 mM NaClO_4_. Cyclic voltammetry was performed between −0.35 V to 0.4 V at a scan rate of 1 V s^−1^. The recorded cyclic voltammograms are depicted in Fig. 4(d). Two voltammetric traces are presented: the purple curve represents the scenario where the droplet size (91 μm radius) was significantly larger than the electrode size, while the red curve corresponds to a smaller droplet where the three-phase boundary approached the electrode surface. The micrographs are labelled as 1 and 2 in Fig. 4(d). Interestingly, when the droplet is large, no redox activity is noted in control experiments. However, when the droplet decreases, we observe the presence of some redox activity at the apparent standard potential of +0.2 V *vs.* Ag/AgCl for the Fe(CN)_6_^3−^/Fe(CN)_6_^4−^ redox couple. Observing a signal from 50 μM is not possible using voltammetry in bulk, which hints at the droplet enhancing mass transfer to the electrode surface, enabling us to detect a signal from K_3_[Fe(CN)_6_]. We propose a mechanism to explain the observations in Fig. 4(e). This could occur due to partial exposure of the electrode surface and the presence of the droplet, which increases the mass-transfer of Fe(CN)_6_^3−^ at the electrode surface, as depicted in the schematic. It's worth noting that in the red trace, we only observe the reduction of Fe(CN)_6_^3−^ to Fe(CN)_6_^4−^, which can now be explained by the Fe(CN)_6_^4−^ generated at the electrode surface escaping to the bulk solution, resulting in the asymmetry in the observed redox activity. At first glance, ultra-low concentration experiments may seem plagued by a significant background signal from K_3_[Fe(CN)_6_] present in the aqueous phase. However, it turns out that the enhanced mass transfer of Fe(CN)_6_^3−^ is beneficial for the system, significantly enhancing the EC′ reaction at the oil|water interface.

## Conclusion

In summary, this study introduces an innovative electrochemical platform capable of detecting and identifying fewer than 1000 molecules of redox analytes. Leveraging the dissolution of (Cp*)_2_Fe^II^ in 1,2-dichloroethane microdroplets within an aqueous continuous phase on a gold microelectrode, we achieved unparalleled sensitivity. By introducing μM amounts of K_3_[Fe(CN)_6_] into the aqueous phase, we initiated a biphasic reaction with (Cp*)_2_Fe^II^, leading to signal amplification through EC′ catalysis when the droplet dimensions were small enough. These results underscore the potential of biphasic reactions combined with dissolving droplets to achieve remarkably low limits of quantitation in electroanalysis. Our platform opens new horizons for ultra-sensitive molecular detection, with broad applications in environmental monitoring and biomedical diagnostics.

## Author contributions

J.H.N and A.R. contributed equally to this work. All authors have agreed to the final version of the manuscript.

## Conflicts of interest

There are no conflicts to declare.

## Supplementary Material

AN-149-D4AN00504J-s001

AN-149-D4AN00504J-s002
