# Supplementary material for: Amplifying the electrochemical footprint of <1000 molecules in a dissolving microdroplet
Source: Analyst. 2024 May 17;149(16):4222–9. doi: 10.1039/d4an00504j (PMC11299638; doi:10.1039/d4an00504j)
Supplement: AN-149-D4AN00504J-s001 [file AN-149-D4AN00504J-s001.pdf]

Electronic Supplementary Material (ESI) for Analyst.
This journal is © The Royal Society of Chemistry 2024

Supplementary Information

Amplifying the Electrochemical Footprint of < 1000 Molecules in a Dissolving Microdroplet

James H. Nguyen[a], Ashutosh Rana[a], and Jeffrey E. Dick[a,b*]

[a]Department of Chemistry, Purdue University, West Lafayette, IN, 47907, USA
[b]Elmore Family School of Electrical and Computer Engineering, Purdue University, West Lafayette, IN, 47907, USA
*Corresponding Author(s)
Jeffrey E. Dick (jdick@purdue.edu)

Table of Contents

Figure or Theory: Description	Page Number
**Figure S1:** Cyclic voltammograms 0.5 mM $(Cp^*)_2Fe^{(II)}$ in an aqueous bulk phase of 200 mM $K_3[Fe(CN)_6]$ in 10 mM $NaClO_4$.	S2

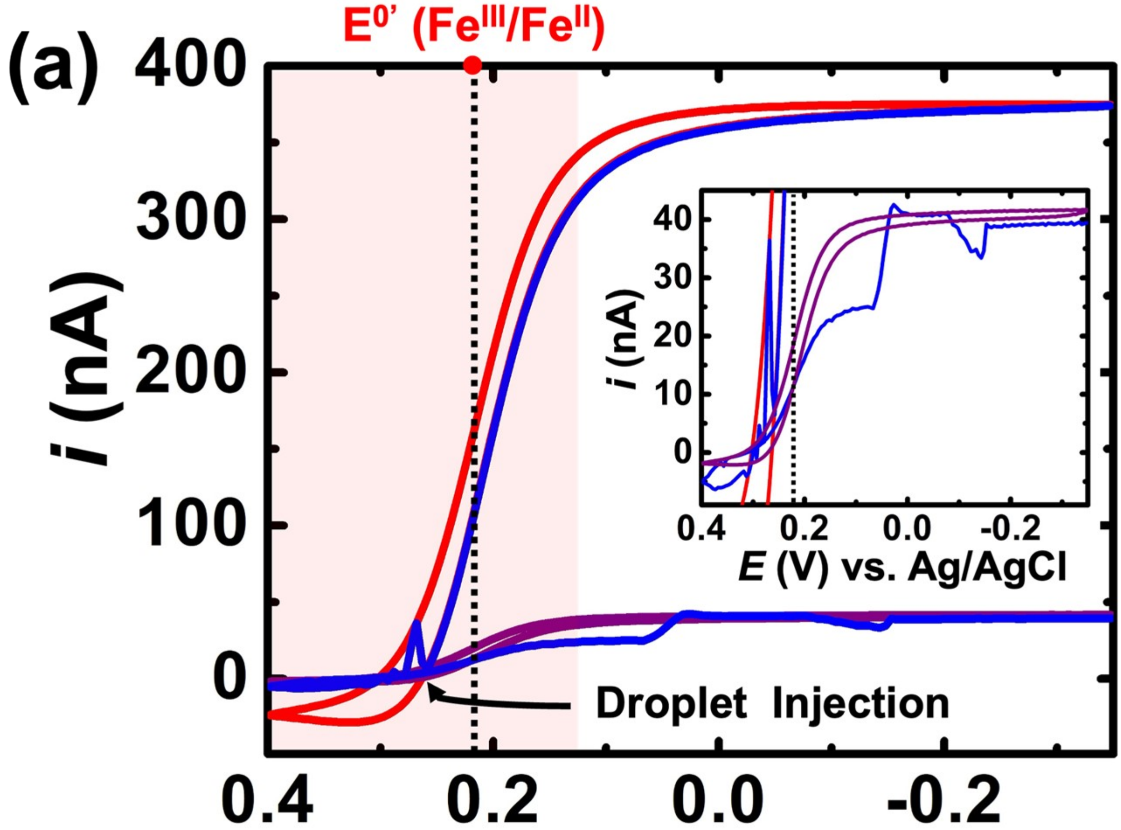

Figure S1. (**a**) Cyclic voltammograms recorded during the dissolution of a DCE droplet containing 0.5 mM $(Cp^*)_2Fe^{(II)}$ in an aqueous bulk phase of 200 mM $K_3[Fe(CN)_6]$ in 10 mM $NaClO_4$. The dashed lines represent the standard apparent potential for the redox couple $Fe(CN)_6^{3-}$ / $Fe(CN)_6^{4}$. Inset (**i**) shows a close up of the purple voltammogram showing suppressed redox activity of $Fe(CN)_6^{3-}$ / $Fe(CN)_6^{4}$ and absence of any signal from Cp_2*$(Fe)^{II}$.
